# Experimental Performance of a Membrane Desorber Operating under Simulated Warm Weather Condensation Temperatures

**DOI:** 10.3390/membranes11070474

**Published:** 2021-06-26

**Authors:** Jonathan Ibarra-Bahena, Wilfrido Rivera, Sandra Daniela Nanco-Mejía, Rosenberg J. Romero, Eduardo Venegas-Reyes, Ulises Dehesa-Carrasco

**Affiliations:** 1 Instituto Mexicano de Tecnología del Agua, Paseo Cuauhnáhuac 8532, Colonia Progreso, Jiutepec 62550, Morelos, Mexico; jibarra@ier.unam.mx (J.I.-B.); eduardo_venegas@tlaloc.imta.mx (E.V.-R.); 2 Instituto de Energías Renovables, Universidad Nacional Autónoma de México, Privada Xochicalco S/N, Col. Centro, Temixco 62580, Morelos, Mexico; wrgf@ier.unam.mx; 3 Facultad de Ciencias Químicas e Ingeniería, Universidad Autónoma del Estado de Morelos, Av. Universidad 1001, Cuernavaca 62209, Morelos, Mexico; me_sany@hotmail.com; 4 Centro de Ingeniería y Ciencias Aplicadas, Universidad Autónoma del Estado de Morelos, Av. Universidad 1001, Cuernavaca 62209, Morelos, Mexico; rosenberg@uaem.mx

**Keywords:** desorption process, air gap membrane distillation, water/LiBr mixture, absorption cooling systems

## Abstract

In absorption systems using the aqueous lithium bromide mixture, the Coefficient of Performance is affected by the desorber. The main function of this component is to separate the refrigerant fluid from the working mixture. In conventional boiling desorbers, constant heat flux and vacuum pressure conditions are necessary to carry out the desorption process, and usually, the absorbers are heavy and bulky; thus, they are not suitable in compact systems. In this study, a membrane desorber was evaluated, operating at atmospheric pressure conditions with a water/lithium bromide solution with a concentration of 49.6% *w*/*w*. The effects of the solution temperature, solution mass flow, and condensation temperature on the desorption rate were analyzed. The maximum desorption rate value was 6.1 kg/m^2^h with the following operation conditions: the solution temperature at 95.2 °C, the solution mass flow at 4.00 × 10^−2^ kg/s, and the cooling water temperature at 30.1 °C. On the other hand, the minimum value was 1.1 kg/m^2^h with the solution temperature at 80.2 °C, the solution mass flow at 2.50 × 10^−2^ kg/s, and the cooling water temperature at 45.1 °C. The thermal energy efficiency, defined as the ratio between the thermal energy used to evaporate the refrigerant fluid with respect to the total thermal energy entering the membrane desorber, varied from 0.08 to 0.30. According to the results, a high solution mass flow, a high solution temperature, and a low condensation temperature lead to an increase in the desorption rate; however, a low solution mass flow enhanced the thermal energy efficiency. The proposed membrane desorber could replace a conventional boiling desorber, especially in absorption cooling systems that operate at high condensation temperatures as in warm weather regions.

## 1. Introduction

As fossil fuel sources are consumed and environmental awareness increases, technologies using renewable or sustainable energy sources are receiving significant interest. In the refrigeration sector, the interest in cooling systems driven by thermal energy is growing [1]. Absorption cooling systems are devices that can operate with renewable thermal energies (such as solar or geothermal) or low-grade waste heat from industrial processes; therefore, they are an eco-friendly option to conventional cooling compression systems. According to Solano-Olivares et al. [2], for the construction of an absorption cooling system, large amounts of energy and materials are required; as a result, the most negative environmental impacts of this technology are focused on this stage. Besides, a currently pending issue is the high ecotoxicity potential which is mostly created by the use of heavy metals for the manufacturing of stainless steel due to the corrosion caused by the saline solutions used as working mixtures. However, the authors concluded that an absorption cooling system powered by solar energy reduces the fossil fuel consumption and greenhouse gases (GHG) emissions by around 80% in both indicators; besides, their waste materials may be almost totally recycled [2]. Despite these ecological advantages, absorption cooling systems are large in size and weight, limiting small-scale applications. Besides, when a heat source is supplied at a low-temperature level, the refrigerant fluid separation from the working mixture (desorption process) is reduced, causing a decrease in the system cooling capacity [3]. Therefore, the desorber (also known as a generator) is the most restrictive component for absorption systems driven by renewable energy sources [4]. Several desorber designs have been proposed to overcome the large volume problem and to improve the heat and mass transfer processes, which include dual components (desorber and condenser) [5,6,7], enhanced falling-film configurations [8,9,10], and microchannel configurations [11,12,13]. In addition to the design geometries, the performance of any desorber is affected by the condenser. The increase in the condenser temperature increases the desorber pressure, which decreases the boiling rate and the Coefficient of Performance (COP) of the whole absorption system [14]. Condensers require auxiliary devices to deliver the latent heat of vaporization to the environment due to the refrigerant fluid condensation. Usually, cooling towers are used; however, they can increase the initial investment and maintenance costs, especially for small-scale applications [15]. In order to reduce the initial cost and avoid water consumption, an air-cooled absorption cooling system could be used [16].

Nonetheless, in the air-cooled mode of operation, the condenser pressure increases as the air temperature increases, causing an increment in the desorption temperature. Besides, in an absorption cooling device using the H_2_O/LiBr mixture, the LiBr crystallization risk increases as the desorption temperature increases [17]. These disadvantages limit the absorption systems for air conditioning in hot weather regions or automotive applications [18]. In this regard, membrane devices have become an alternative to conventional desorbers. Membrane Distillation (MD) is a separation process driven by thermal energy, mostly applied to separate volatile dissolved substances from a solvent/solute mixture [19]. The main advantages of MD, with respect to the conventional boiling desorption, include: operating temperatures lower than the boiling point temperatures of working mixtures, operating pressures close to the atmospheric condition, compact components, and by using polymeric materials, corrosion problems can be avoided [20]. In recent years, membrane-based devices have been analyzed and proposed to replace the conventional boiling desorbers, particularly for absorption cooling systems using the H_2_O/LiBr mixture. Table 1 shows the experimental reports about these components. Other aqueous mixtures have been tested [21,22], as well as different membrane-based desorber operation modes [23]. Comprehensive literature reviews about membrane technology in absorption heat pumps were carried out by Asfand and Bourouis [24] and Ibarra-Bahena et al. [25]. 

From the data shown in Table 1, the condensation temperature range used in membrane-based desorbers has been below 30 °C; in this paper, an experimental evaluation of a hydrophobic membrane-based desorber with condensation temperatures up to 45 °C at atmospheric pressure conditions is presented. The Air Gap Membrane Distillation (AGMD) configuration was used in the membrane device. The effects of different parameters such as H_2_O/LiBr solution temperature, cooling water temperature, and solution mass flow on the desorption rate have been analyzed as well on the thermal energy efficiency. The aim of this paper is to demonstrate the technical feasibility of the membrane desorber operated at condensation temperatures up to 45 °C for absorption cooling systems.

## 2. Air Gap Membrane Distillation Process

**Figure 1:**
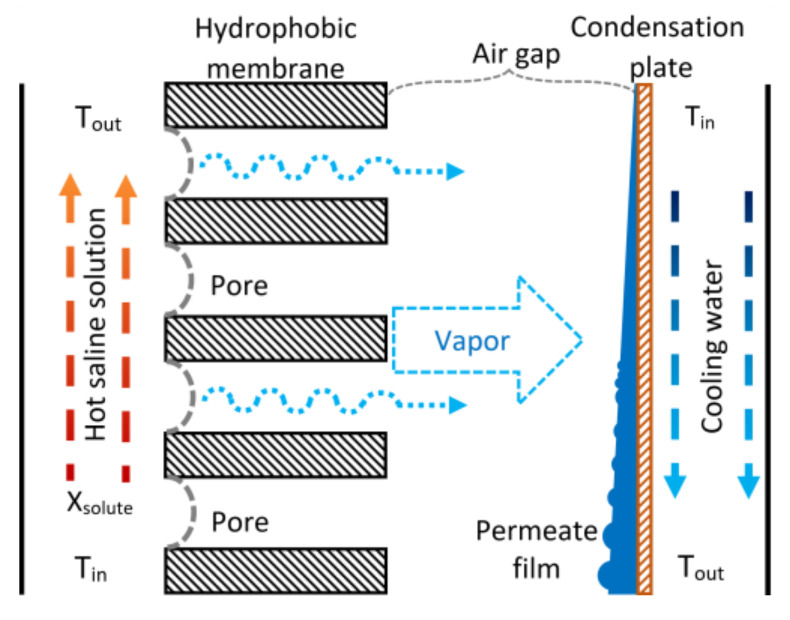
Schematic diagram of the AGMD process.

Membrane distillation is a thermal separation process in which only water vapor (or other volatile molecules) crosses through a hydrophobic porous membrane. An amount of water is evaporated on the liquid-membrane interphase at the hot side, passing through the porous membrane, and condensed on the cold side. The temperature difference between both membrane sides generates a partial pressure difference; this is the mass transfer driving force. The membrane’s function is to hold the vapor/liquid interphase created on both sides and, due to its hydrophobic nature, only water vapor crosses through it [34]. There are at least four configurations to generate the partial pressure difference across the membrane: Direct Contact Membrane Distillation (DCMD), where the hot saline solution and the permeate (that works as cooling fluid) are in direct contact with each side of the membrane surface; Sweeping Gas Membrane Distillation (SGMD), where an inert gas is used to transport the vapor to the cold side to condense outside the membrane module; Vacuum Membrane Distillation (VMD), where vacuum is generated in the cold side with a vacuum pump; and Air Gap Membrane Distillation (AGMD), where a stagnant air layer separates the membrane and the condensation surface; in the SGMD and VMD configurations, the permeate vapor condensation occurs in an external condenser [35]. An AGMD module comprises three sections: the feed section, the air-gap or permeate section, and the cooling section (see Figure 1). 

The vapor coming from the feed section diffuses into the air gap, and it condenses on the cooling plate; the condensed permeate is drained out of the module. The coolant fluid flows on the other side of the cooling plate, removing the heat load produced by the vapor condensation. The AGMD configuration reduces the heat loss, and the temperature polarization is lower due to the stagnant air gap (because air has low thermal conductivity), but simultaneously, an additional mass transfer resistance is created. This mass transfer resistance reduces the permeate flux, and it is directly proportional to the airgap width [36]. A remarkable advantage of AGMD modules for absorption cooling applications is that they can operate at atmospheric pressure conditions. 

## 3. Methodology

Different solution temperatures, solution mass flow rates, and cooling water temperatures were varied to analyze their effect on the desorption process. The H_2_O/LiBr solution was selected since it is the most used solution mixture in absorption cooling systems (using water as working fluid), and in general, the desorption process takes place at temperatures lower than 100 °C [37,38]. With the experimental data, a thermal energy efficiency analysis was carried out to quantify the membrane desorber’s performance.

### 3.1. Experimental Setup

The membrane-based desorber was integrated by two support plates made of Nylamid with 300 mm length, 200 mm wide, and 25.4 mm thickness; neoprene gaskets and thermal-resistant silicon gaskets with 1 and 3 mm thickness, respectively; a metallic mesh to upholding the membrane; an aluminum cooling plate with 0.4 mm thickness; and 24 bolts and nuts. The features of the membrane used are shown in Table 2. The desorber membrane area used in the desorber was 144 cm^2^. The experimental tests were carried out in Temixco, Morelos, Mexico, where the atmospheric pressure is approximately 87 kPa.

**Figure 2:**
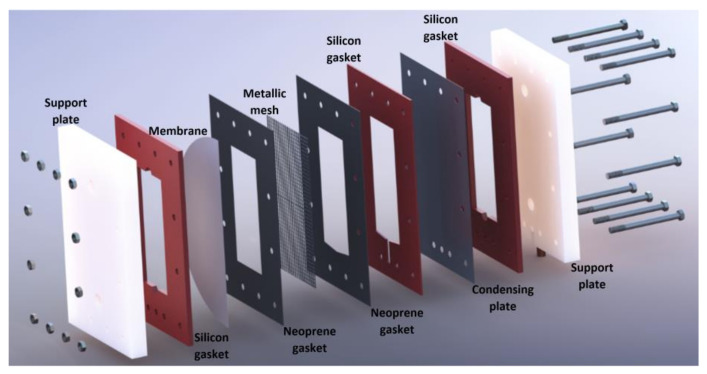
Exploded view of the experimental membrane-based desorber.

The H_2_O/LiBr solution channel was 180 mm in length, 80 mm wide, and 3 mm thickness, and it was created by the junction of the support plate, the thermal-resistant silicon gasket, and the hydrophobic membrane. An air gap with 3 mm of thickness was at the other side of the hydrophobic membrane, two neoprene gaskets, the metallic mesh, one silicon gasket, and one side of the condensing plate. Finally, the cooling water flowed in the channel created by the other side of the condensing plate, the thermal-resistant silicon gasket, and the support plate. Figure 2 shows the components of the experimental membrane-based desorber.

**Figure 3:**
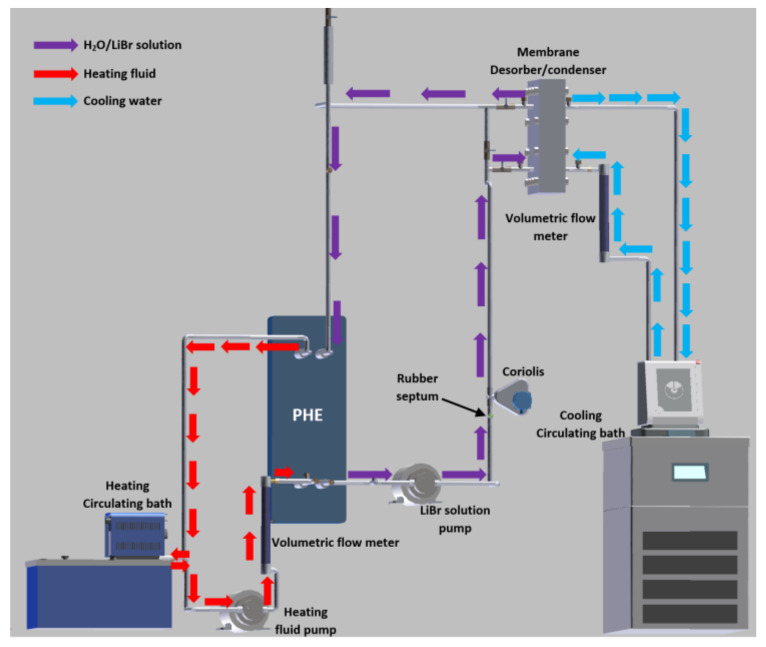
Schematic diagram of the experimental setup.

**Figure 4:**
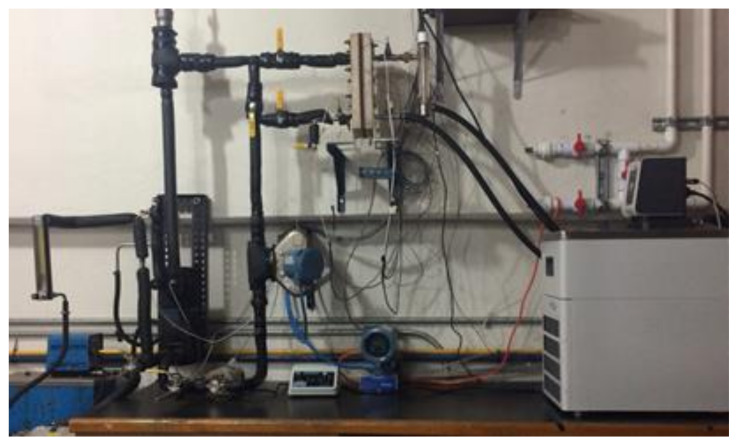
Photograph of the experimental setup.

A 316 SS plate heat exchanger (PHE) was used to heat transfer from the heating fluid to the H_2_O/LiBr solution, and the thermal source was a Cole-Parmer Polystat digital heating circulating bath of 6 L volume and 1000 W electrical power. The cooling water temperature was kept constant with a Julabo CORIO 601F refrigerated/heating circulator bath of 10 L volume and 2000 W. A Coriolis mass flowmeter was used to measure the solution mass flow rate (*ṁ_LiBr_*). The heating fluid stream (*V_hf_*) and the cooling water stream (*V_cw_*) were measured with analogical flowmeters. A rubber septum was placed in the LiBr solution loop, and a liquid sample of the H_2_O/LiBr solution was drawn out for each experimental test. An ABBEMAT 200 refractometer was used to determine the salt solution concentration using a refractive index (RI) correlation previously reported for H_2_O/LiBr mixture [39]. The solution and the heating fluid were pumped by using two gear pumps of 32 W power. An electronic weighing scale was used to measure the amount of distilled water (*w_dis_*). The temperatures of the inlet and outlet streams of the membrane-based desorber and the PHE were measured with RTD pt100 temperature sensors. The experimental temperatures and the solution mass flow data were recorded by an Agilent data acquisition unit. Figure 3 describes the experimental setup, while Figure 4 shows a photograph of the experimental setup.

The uncertainties of the measured variables are shown in Table 3, while the operating conditions are present in Table 4. 

### 3.2. Thermal Energy Efficiency Analysis

As was described in previous sections, the AGMD process is driven by thermal energy to heat the aqueous solution. The thermal energy efficiency (*η_T_*) is defined as the ratio between the heat used to evaporate part of the refrigerant fluid from the solution and the heat supplied to the AGMD device [40]:

(1) $$\eta_{T}=\frac{Effective\;heat\;for\;evaporation}{Thermal\;energy\;input}$$

In the AGMD module analyzed, Equation (1) was written as:

(2) $$\eta_{T}=\frac{J_{w}\lambda_{vap}A_{mem}}{{\dot{m}}_{LiBr}Cp_{LiBr}\left(T_{LiBr,\;in}-T_{LiBr,\;out}\right)}$$

where *λ_vap_* is the water vaporization heat, *A_mem_* is the membrane area, *ṁ_LiBr_* is the H_2_O/LiBr solution mass flow, *Cp_LiBr_* is the H_2_O/LiBr solution specific heat, and *T_LiBr,in_* and *T_LiBr,out_* are the H_2_O/LiBr solution inlet and outlet temperatures, respectively. 

The desorption rate (*J_w_*) is defined as the mass flow of water vapor desorbed (*ṁ_vap_*) crossing the membrane area.

(3) $$J_{w}=\frac{{\dot{m}}_{LiBr}}{A_{mem}}$$

The thermodynamic properties of the H_2_O/LiBr solution were calculated using the correlations reported by Kaita [41].

## 4. Results

### 4.1. Desorption Rate

**Figure 5:**
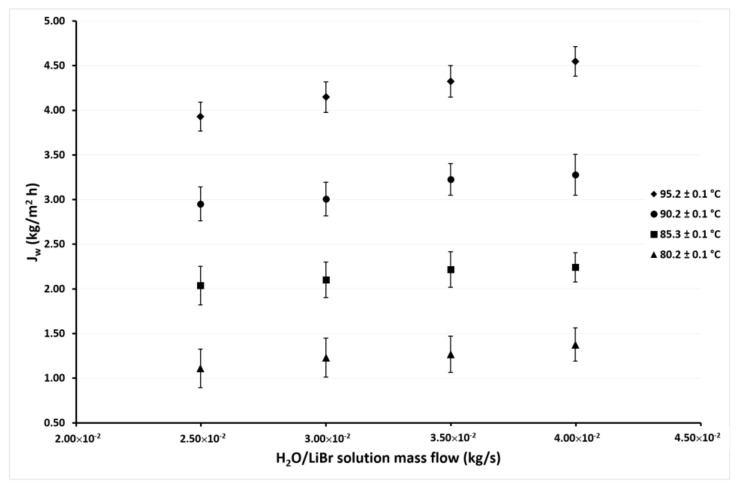
Desorption rate as a function of the *ṁ_LiBr_* with *T_cw,in_* = 45.1 ± 0.1 °C.

**Figure 6:**
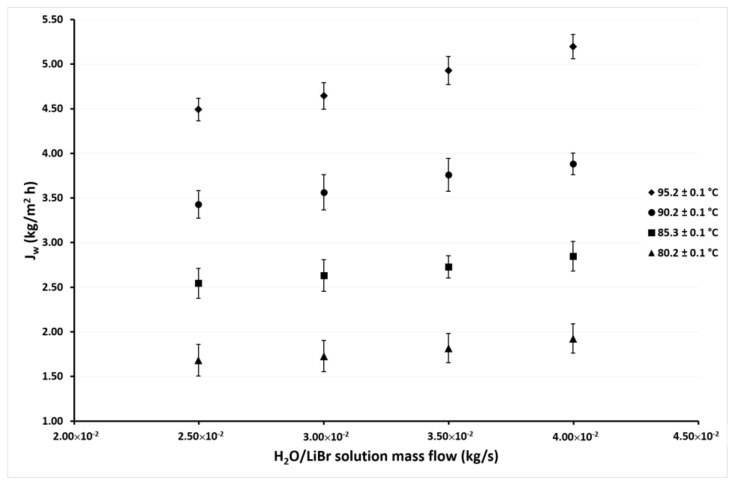
Desorption rate as a function of the *ṁ_LiBr_* with *T_cw,in_* = 40.1 ± 0.1 °C.

**Figure 7:**
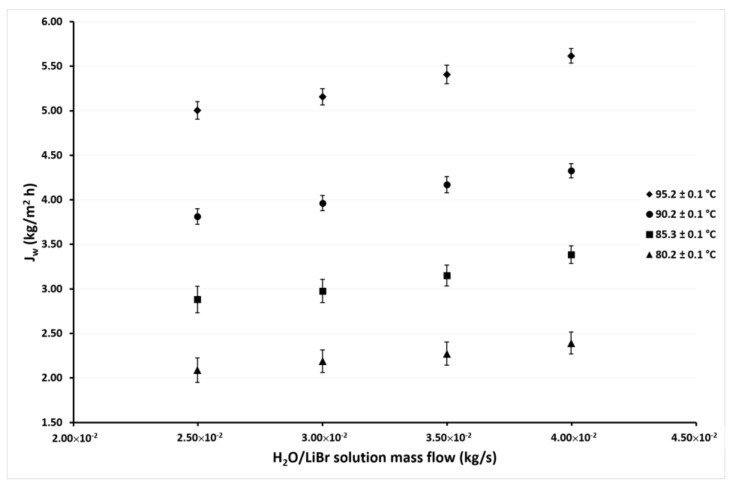
Desorption rate as a function of the *ṁ_LiBr_* with *T_cw,in_* = 35.1 ± 0.1 °C.

**Figure 8:**
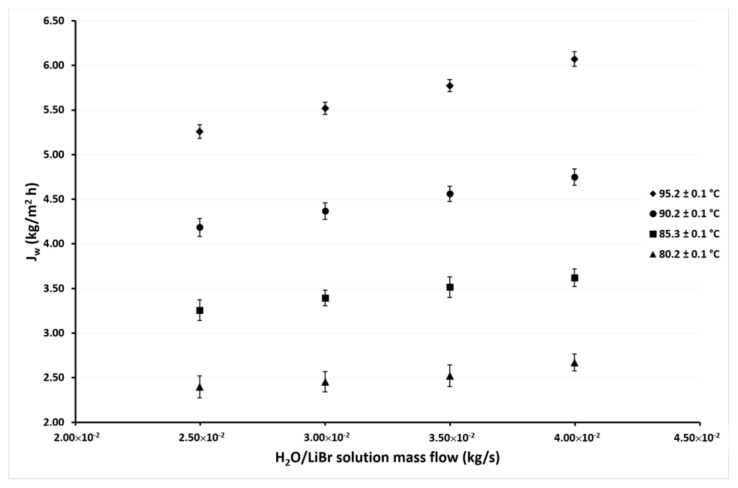
Desorption rate as a function of the *ṁ_LiBr_* with *T_cw,in_* = 30.1 ± 0.1 °C.

The experimental desorption rate (*J_w_*) was measured based on the mass of distillate water (*w_dis_*) produced by the membrane-desorber after a defined time period. The *J_w_* as a function of the H_2_O/LiBr solution mass flow rate (*ṁ_LiBr_*) at different inlet H_2_O/LiBr solution temperature (*T_LiBr,in_*) and different inlet cooling water temperatures (*T_cw,in_*) are shown in Figure 5, Figure 6, Figure 7 and Figure 8. *T_LiBr,in_* and *T_cw,in_* refer to the temperature of the H_2_O/LiBr solution and the cooling water, respectively, at the membrane-desorber entrance. Figure 5 shows the desorption rate as a function of the solution mass flow rate with solution temperatures of 80 °C, 85 °C, 90 °C, and 95 °C, and constant cooling water temperature of 45.1 °C. It can be observed that the effect of the solution temperature is higher than the solution mass flow effect on the desorption rate. The increment of *ṁ_LiBr_* from 2.50 × 10^−2^ kg/s to 4.00 × 10^−2^ kg/s causes a moderate increment (15% on average) on *J_w_* with constant *T_LiBr,in_*. However, *J_w_* considerably raises (240% on average) at the highest *T_LiBr,in_* value of 95.2 °C, with respect to the lowest temperature of 80.2 °C, with constant *ṁ_LiBr_.* This behavior was expected, since, as it was commented in Section 2, the AGMD is a thermal separation process, and the driving force is the partial pressure difference, which is related to the temperature difference between both sides of the membrane; therefore, the increment in the solution temperature increases the partial pressure of the water vapor (refrigerant fluid) [42]. The solution mass flow rate enhances the desorption rate because, as the solution velocity increases, the heat and mass transfer resistances are reduced at the solution-membrane interphase [26,43]; however, the thermophysical properties of the H_2_O/LiBr which affect the heat and mass transfer resistances are influenced by the solution temperature.

Figure 6 shows the variation of *J_w_* as a function of *ṁ_LiBr_* with the same solution temperatures as in Figure 5 but with the cooling water temperature of 40.1 °C. As can be seen, the desorption rate behavior is similar as shown in Figure 5, but in this case, the *J_w_* values are higher, since in this case, the minimum value was 1.7 kg/m^2^h, and the maximum value was 5.2 kg/m^2^h. These values represent an increment of 52% and 14% of the minimum and maximum values, respectively, over those reported in Figure 5 at the cooling water temperature of 45.1 °C. The effect of the *ṁ_LiBr_* was similar to that observed in Figure 5, since the increment of *J_w_* was 14% on average at the highest *ṁ_LiBr_* value (4.00 × 10^−2^ kg/s) with respect to the lowest value of 2.50 × 10^−2^ kg/s at constant a *T_LiBr,in_*. Meanwhile, the *J_w_* increased 169% on average at *T_LiBr,in_* = 95.2 °C with respect to the *T_LiBr,in_* = 80.2 °C.

Figure 7 and Figure 8 show the variation of *J_w_* as a function of *ṁ_LiBr_* at cooling water temperatures of 35.1 °C and 30.1 °C, respectively. In these figures, it can be seen that the same behaviors were shown in the previous figures. The effect of the *ṁ_LiBr_* was the same magnitude as observed in Figure 5 and Figure 6 since the desorption rate increased by 14% and 13% on average at *T_cw_* = 35.1 °C and *T_cw_* = 30.1 °C, respectively. When *T_LiBr,in_* raised from 80.2 °C to 95.2 °C, the *J_w_* increased 137% and 125% on average, respectively. 

In Figure 5, Figure 6, Figure 7 and Figure 8, it can also be observed that the *J_w_* values continuously increase as the condenser temperature decreases due to the mass transfer driving force increased by the reduction of the vapor partial pressure in the membrane desorber cooling plate (cold side). This effect was remarkable at *T_LiBr,in_* = 80.2 °C since the *J_w_* increased 164% on average at *T_cw,in_* = 30.1 °C, with respect to *T_cw,in_* = 45.1 °C. The solution temperature effect was the most influential parameter on the desorption rate, the cooling water temperature was the second, and finally, the solution mass flow. These results are in concordance with the literature, since for the membrane desorber operation, the highest technically viable solution temperature is recommended [44].

### 4.2. Thermal Energy Efficiency

**Figure 9:**
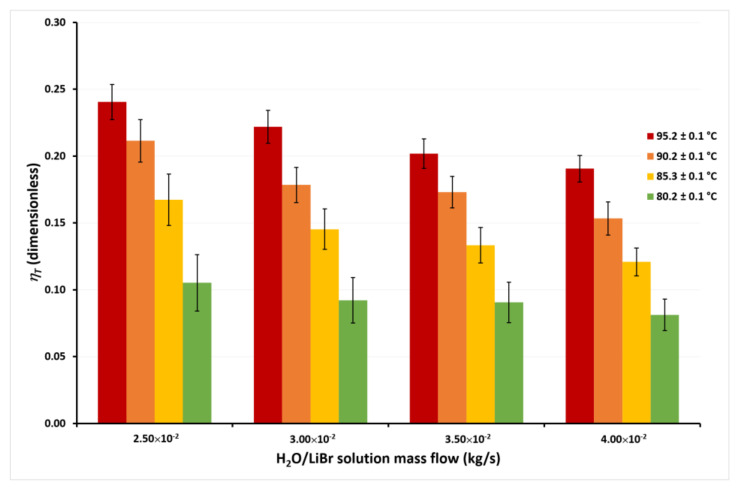
Thermal energy efficiency as a function of the *ṁ_LiBr_* with *T_cw,in_* = 45.1 ± 0.1 °C.

**Figure 10:**
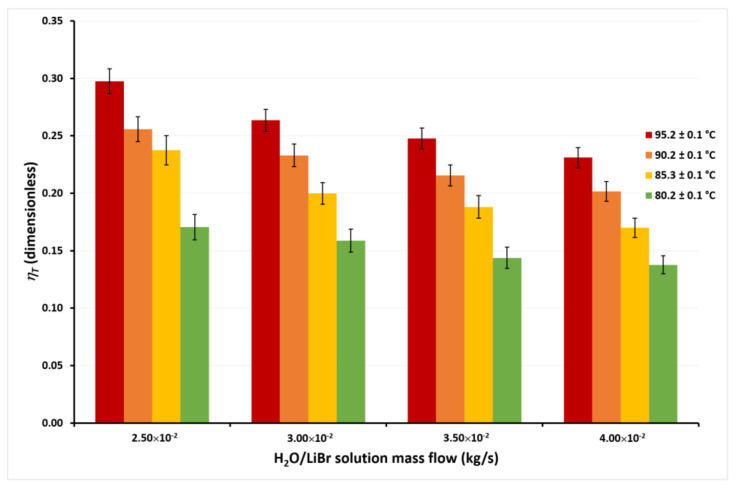
Thermal energy efficiency as a function of the *ṁ_LiBr_* with *T_cw,in_* = 30.1 ± 0.1 °C.

The calculated energy efficiencies (*η_T_*) for the highest (*T_cw,in_* = 45.1 °C) and lowest (*T_cw,in_* = 30.1 °C) cooling water temperatures for the tested H_2_O/LiBr solution mass flows and the different solution temperatures are shown in Figure 9 and Figure 10. Similar behavior of the *η_T_* was calculated with the other cooling water temperatures, but they are not presented, in an attempt to avoid repeatability. 

In Figure 9, it can be seen that the *η_T_* achieves the highest values at the lowest *ṁ_LiBr_* value, independently of the solution temperatures. This occurs since, as previously discussed, although *ṁ_LiBr_* increased 60%, from 2.50 × 10^−2^ kg/s to 4.00 × 10^−2^, *J_w_* increased by only 14% on average at any *T_LiBr,in_* value. Therefore, an increase in the solution mass flow causes a net decrease in the *η_T_* from Equation (2). On the other hand, it can be observed that *η_T_* increased 132% on average for the different mass flows when *T_LiBr,in_* increases from 80.2 °C to 95.2 °C. This is because, as observed in Figure 5, Figure 6, Figure 7 and Figure 8, the effect of the solution temperature on the *J_w_* was higher than the effect of the mass flow. The effect of the cooling water temperature on the thermal energy efficiency can be appreciated by comparing the *η_T_* values from Figure 9 and Figure 10. At a solution temperature of 80.2 °C, a mass flow of 4.00 × 10^−2^ kg/s, and a condenser temperature of 45.1 °C, the *η_T_* is 0.08, while at the same values of the solution temperature and the solution mass flow but a condenser temperature of 30.1 °C, the *η_T_* is 0.14. Taking into account all the *η_T_* values at the different mass flows, the efficiency raised 66% on average at a *T_LiBr,in_* = 80.2 °C, when the condenser temperature decreases from 45.1 °C to 30.1 °C. However, at the highest solution temperature (*T_LiBr,in_* = 95.2 °C) the *η_T_* increased only 22% (average) at *T_cw_,_in_* = 30.1 °C, with respect to *T_cw,in_* = 45.1 °C. 

According to Figure 4, Figure 5, Figure 6 and Figure 7, the desorption rate is slightly affected by the cooling water temperature compared to the solution temperature. As the cooling water temperature increases, the desorption rate decreases by the reduction in the vapor pressure gradient. The vapor partial pressure in the hot side increases exponentially with the increase in the solution temperature compared to the small increment of the vapor partial pressure by the cooling water reduction [45]; thus, the effect of the cooling water temperature on the thermal efficiency was higher at *T_LiBr,in_* = 80.2 °C than *T_LiBr,in_* = 95.2 °C. For this reason, some authors suggest using the highest available solution temperature rather than decreasing the cooling water temperature to improve the performance of a membrane distillation device [36,44]. The highest *η_T_* value was 0.30 at *T_LiBr,in_* = 95.2 °C, *T_cw,in_* = 30.1 °C, and *ṁ_LiBr_* = 2.50 × 10^−2^ kg/s, while the lowest *η_T_* value was 0.08 at *T_LiBr,in_* = 80.2 °C, *T_cw,in_* = 45.1 °C, and *ṁ_LiBr_* = 4.00 × 10^−2^ kg/s.

The calculated thermal energy efficiency for the desorption process was significantly lower than the conventional desalination process, which is from 0.95 to 0.99 [42,46,47]. However, when concentrated salt solutions (like brines) are used, the *η_T_* decreases; e.g., with a 24% *w*/*w* of NaCl concentration, the *η_T_* was 0.30 [48].

## 5. Conclusions

A membrane-based desorber using the AGMD configuration operating with the H_2_O/LiBr mixture at atmospheric pressure conditions was evaluated. Condenser temperatures from 30 to 45 °C were selected to simulate environment temperatures in warm weather regions. The solution temperature, the cooling water temperature, and the solution mass flow on the desorption rate were studied. Since membrane distillation is a thermal separation process, the desorption rate was improved as the LiBr solution temperature increased and the cooling water temperature decreased. Additionally, according to the experimental data, as the LiBr solution mass flow increased, the desorption rate increased also. It can be explained by the decrease in the heat and concentration boundary layers as the solution velocity flow increased; thus, the desorption rate was enhanced. However, the influence of the solution mass flow on the desorption rate was minimal (14% on average) with respect to the cooling water and solution effects. The maximum desorption rate value was 6.1 kg/m^2^ h at a solution mass flow of 4.00 × 10^−2^ kg/s, solution temperature of 95.2 °C, and a cooling water temperature of 30.1 °C. On the other hand, the minimum value was 1.1 kg/m^2^h, and it was obtained at the lowest mass flow and solution temperature and the highest cooling water temperature. Unlike the desorption rate, the thermal energy efficiency was improved as the solution mass flow decreased. The highest value was 0.30 at the highest solution temperature and the lowest cooling water temperature and solution mass flow. The membrane desorber proposed in this study has feasible potential in absorption cooling applications, especially with systems operating at high condense temperatures (up to 45 °C) as those required in warm weather regions. In addition, the desorption process was carried out at atmospheric pressure conditions; therefore, a vacuum pump was unnecessary for the operation of this component, which is an advantage with respect to the conventional boiling desorbers. 

## Figures and Tables

**Table 1:** Experimental operating conditions of the membrane-based desorbers reported in the literature.

Authors	Configuration	*d_p_* (μm)	*X_LiBr_* (% *w*/*w*)	*T_LiBr_* (°C)	*T_Con_* (°C)	*ṁ_LiBr_* (kg h^−1^)	*J_w_* (kg m^−2^ h^−1^)
Venegas et al. [26]	Flat sheet	0.45	45.8	58 to 60	25.7	0.5 to 1.7	5.8 to 15.1
Ibarra et al. [27]	Flat sheet	0.22	49.8	75.2 to 95.3	14.4 to 25.4	90.0	1.5 to 5.7
Hong et al. [28]	Hollow fiber	0.16	51 to 58	65 to 83	NA	173 to 269	0.4 to 3.4
Ibarra et al. [29]	Flat sheet	0.45	45.7 to 58.7	74.4 to 95.9	15.6 to 20.0	58.7 to 90.0	0.3 to 9.7
Isfahani et al. [30]	Flat sheet	0.45	48 to 51	50 to 125	NA	0.75 to 3.25	0.0 to 37.8
Bigham et al. [31]	Flat sheet	1.00	48	50 to 125	NA	2.5	0.0 to 34.2
Wang et al. [32]	Hollow fiber	0.16	50	65 to 88	NA	40 to 120	0.3 to 2.0
Sudoh et al. [33]	Fl at sheet	0.20	35 to 55	35 to 100	15	NA	1.8 to 18

**Table 2:** Membrane features.

Material	PTFE (Polytetrafluoroethylene)
Mean pore diameter (*d_p_*)	0.22 μm
Porosity (*φ*)	70%
Thickness (*δ_m_*)	175 μm

**Table 3:** Uncertainty of the measured variables.

Variable	Sensor/Instrument	Operation Range	Uncertainty
Temperature (*T*)	RTD PT100	−30 to 350 °C	± 0.1 °C
Volumetric flow (*V_cw_*)	Volumetric flowmeter	0 to 7 L/min	± 5.0% f.s. *
Volumetric flow (*V_hf_*)	Volumetric flowmeter	0 to 1.2 L/min	± 4.0% f.s. *
Mass flow *(m_LiBr_*)	Coriolis mass flowmeter	0 to 4.0 × 10^−^^2^ kg/s	± 0.1%
Distillate water weight *(w_dis_*)	Electronic balance	0 to 600 g	± 0.01 g
Refractive index (*RI*)	Electronic refractometer	1.3000 to 1.7200	± 0.0001

* f.s., full scale.

**Table 4:** Experimental operating conditions.

Parameter	Value
LiBr concentration (% *w*/*w*)	49.61 ± 0.07
Cooling water volumetric flow (L/min)	2.0 ± 0.35
H_2_O/LiBr solution mass flow (kg/s)	2.50 × 10^−2^ ± 2.22 × 10^−5^
	3.00 × 10^−2^ ± 3.10 × 10^−5^
	3.50 × 10^−2^ ± 2.57 × 10^−5^
	4.00 × 10^−2^ ± 2.44 × 10^−5^
LiBr solution temperature (°C)	95.2 ± 0.1
	90.2 ± 0.1
	85.3 ± 0.1
	80.2 ± 0.1
Cooling water temperature (°C)	45.1 ± 0.1
	40.1 ± 0.1
	35.1 ± 0.1
	30.1 ± 0.1

## Data Availability

Not applicable.
